# The role and mechanisms of Microglia in Neuromyelitis Optica Spectrum Disorders

**DOI:** 10.7150/ijms.61153

**Published:** 2021-06-16

**Authors:** Wenqun Li, Jiaqin Liu, Wei Tan, Yedi Zhou

**Affiliations:** 1 Department of Pharmacy, The Second Xiangya Hospital, Central South University, Changsha, 410011, Hunan, China.; 2 Institute of Clinical Pharmacy, Central South University, Changsha, 410011, Hunan, China.; 3 Department of Ophthalmology, The Second Xiangya Hospital, Central South University, Changsha, Hunan 410011, China.; 4 Hunan Clinical Research Center of Ophthalmic Disease, Changsha, Hunan 410011, China.

**Keywords:** microglia, neuromyelitis optica spectrum disorder, demyelination, microglial activation

## Abstract

Neuromyelitis optica spectrum disorder (NMOSD) is an autoimmune neurological disease that can cause blindness and disability. As the major mediators in the central nervous system, microglia plays key roles in immunological regulation in neuroinflammatory diseases, including NMOSD. Microglia can be activated by interleukin (IL)-6 and type I interferons (IFN-Is) during NMOSD, leading to signal transducer and activator of transcription (STAT) activation. Moreover, complement C3a secreted from activated astrocytes may induce the secretion of complement C1q, inflammatory cytokines and progranulin (PGRN) by microglia, facilitating injury to microglia, neurons, astrocytes and oligodendrocytes in an autocrine or paracrine manner. These processes involving activated microglia ultimately promote the pathological course of NMOSD. In this review, recent research progress on the roles of microglia in NMOSD pathogenesis is summarized, and the mechanisms of microglial activation and microglial-mediated inflammation, and the potential research prospects associated with microglial activation are also discussed.

## Introduction

Neuromyelitis optica (NMO) is a demyelinating autoimmune disease in which the optic nerves and spinal cord are most commonly involved 1, and was first described by Eugène Devic's team in 1894 2. Some NMO patients will eventually become blind and disabled that even need to be wheelchair-bound 3, which will greatly reduce quality of life and cause a great economic burden.

Similar to multiple sclerosis (MS), NMO is also an inflammatory demyelinating disease. However, in NMO, astrocytes are the targets of immune attack, rather than oligodendrocytes, as occurs in in MS. These two diseases could be reliably distinguished by the detection of the biomarker aquaporin-4 (AQP4)-IgG, which is present in the majority of NMO patients but usually absent in cases of MS 4. On this basis, the concept of neuromyelitis optica spectrum disorder (NMOSD) was first proposed and defined in 2007 5. In 2015, the latest diagnostic guidelines included NMO in NMOSD and divided NMOSD into AQP4-IgG-positive and AQP4-IgG-negative forms according to the expression of the AQP4 antibody 6. In addition to AQP4-IgG, another biomarker, myelin oligodendrocyte glycoprotein (MOG)-IgG, has been identified and used for the diagnosis of NMOSD; AQP4-IgG has been detected in approximately 70% of NMOSD patients globally, and MOG-IgG has been detected in approximately 40% of AQP4-Ab-negative NMOSD patients 7. According to many epidemiological studies, NMOSD has great importance in the prevalence in different regions and racial groups worldwide 8, 9. Although the prevalence of NMOSD is limited, severe autoimmune attacks may cause spinal cord and optic nerve involvement, which leads to paralysis and blindness in the patients 10, 11.

Immunosuppressive therapies have been used to prevent relapses and decrease attack severity in patients with NMOSD 12. For instance, because the repopulation of B cells is relevant to the relapse rate of NMOSD, immunotherapies targeting B cells and related proteins were demonstrated to be effective in treating NMOSD 13, 14, and several drugs have been licenced and applied as clinical therapies 15, 16. In addition to B cell depletion therapy, numerous kinds of treatments have been demonstrated to be effective in treating NMOSD, such as interleukin (IL)-6 receptor antagonists, complement blockers and other monoclonal antibodies (such as aquaporumab, bevacizumab and ublituximab) 17. The efficacy of novel therapies (including eculizumab to target the complement system, satralizumab to target the IL-6 receptor, and inebilizumab to target B cells) has been assessed by randomized controlled trials of patients with NMOSD, and the results show that these therapies were beneficial in preventing autoimmune attacks, but the studies also show various efficacy, safety, tolerability, and practical considerations associated with these drugs 18.

As an essential inflammatory cytokine, IL-6 has been reported to be highly expressed in the serum and cerebrospinal fluid (CSF) of NMO patients, and IL-6 levels were positively associated with disease severity 19. It is reported that Th1 and Th17 cytokines/chemokines are upregulated in NMO 20, and the dominant Th17-related response in NMO patients is positively correlated with neurological disability 21. Moreover, the serum concentrations of CXCL6/GCP-2, Midkine and C5/C5a are significantly altered between AQP4-positive NMO and MOG-positive NMO cases, which indicates different immunological mechanisms of NMO pathogenesis that are associated with these two kinds of antibodies 22.

As native macrophages in the central nervous system (CNS), microglia plays diverse roles and has phagocytic and pro- and anti-inflammatory functions in numerous neurological diseases 23, 24. Microglia also plays a key role in the immunoregulation of neuroinflammatory diseases 25. However, in recent years, investigations on NMO have often focused on astrocytes, while the regulatory roles of microglia have been largely neglected 26. Histopathological analysis demonstrates prominent infiltration and activation of macrophages/microglia in NMO lesions 27. Microglia are the major mediators of CNS inflammation, and AQP4-IgG can induce cytokine production by activating astrocytes and lead to bystander activation towards microglia 28. As one of the major antibody-mediated effects of NMOSD, microglial activation has both protective and disruptive effects, while the regulation of these two opposing effects remains to be further studied 29. Chen et al*.* indicate that with the involvement of complement, astrocyte-microglia crosstalk plays a critical role in promoting the development of NMO 30. Therefore, microglia are thought to be potential therapeutic targets in treating NMO 26.

In this review, we summarize the recent research progress on the roles of microglia in NMOSD pathogenesis, and the mechanisms of microglial activation and microglial-mediated inflammation are also discussed. We also suggest the potential research prospects of microglial activation in the context of basic research and clinical practice.

## The upstream mechanism of microglial activation in NMOSD

### IL-6

Activated microglia can recruit macrophages and neutrophilic granulocytes to the NMO lesions, induce the production of IL-1β, IL-6, IL-17, and high-mobility group box 1 protein (HMGB1), which might be involved in NMO pathogenesis 31. IL-17/IL-8 axis activation in cerebrospinal fluid (CSF) and the increase in HMGB1 in the plasma of NMOSD patients have been determined 32, 33. More importantly, CSF levels of IL-6 in NMOSD patients are much higher compared to MS patients, and there is strong evidence showing that IL-6 may play a key role in the pathogenesis of NMOSD 34.

**Figure 1:**
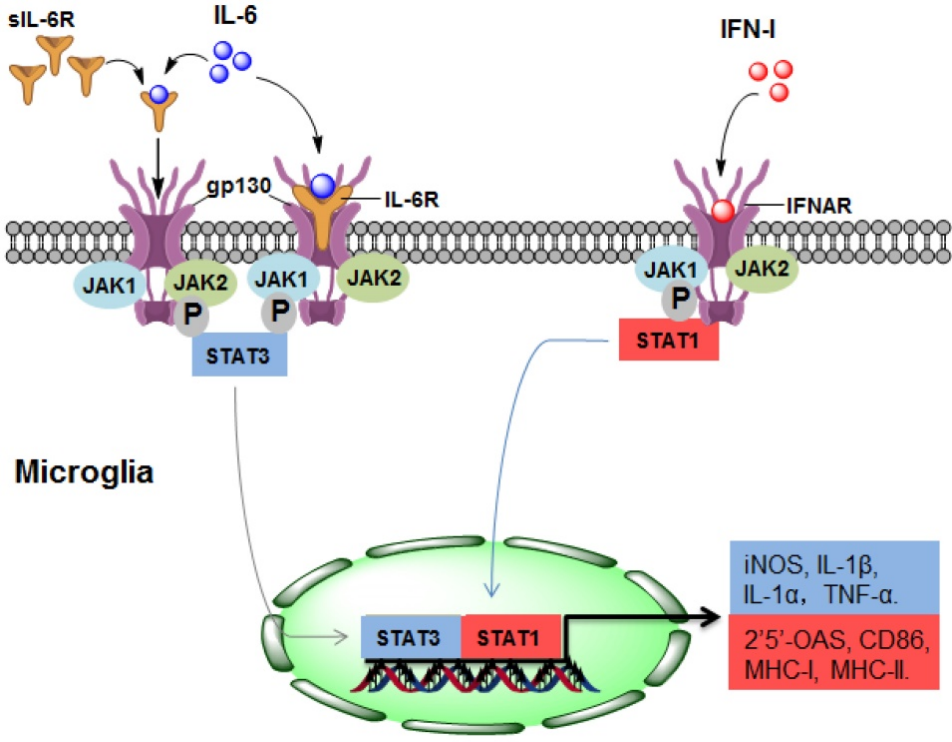
** The upstream mechanism of microglial activation in NMOSD.** (1) IL-6 activates microglia by directly binding to IL-6R/gp130 (classical signalling) or sIL-6R (trans-signalling), leading to STAT3 activation and ultimately promoting the gene expression of iNOS, IL-1α, IL-1β and TNF-α. (2) In response to IFN-I, STAT1 is activated in microglia, resulting in the gene expression of 2'5'-OAS, CD86, MHC-I and MHC-II. IL-6, interleukin-6; IFN-I: type I interferon; sIL-6R, soluble IL-6R; JAK, Janus kinase; STAT, signal transducer and activator of transcription; MHC, major histocompatibility complex.

As a potent regulator of cellular communication, the inflammatory factor IL-6 is responsible for both innate and adaptive inflammatory responses by interacting with IL-6 receptor-α (IL-6R) 35. Many CNS-resident cells can produce IL-6, but IL-6R is expressed on a limited subset of cells. IL-6R is mainly found on the surface of microglia but not on the surface of astrocytes, oligodendrocytes, endothelial cells, or neurons 36. IL-6 binds to membrane-bound IL-6R on microglia, triggering gp130 homodimerization to form a functional receptor complex. The homodimerization of the receptor complex activates Janus kinase 1 (JAK1) and JAK2, leading to the activation of signal transducers and activators of transcription (STAT) 3 37. Thus, activated STAT3 may translocate into the nuclei and mediate the expression of IL-6-regulated genes (Figure 1).

During the pathogenesis of NMOSD, the promoting effect of IL-6 may be mainly due to IL-6-mediated activation of microglia through binding with IL-6R. In CNS cells lacking IL-6R, soluble IL-6R (sIL-6R) mediates the response to IL-6, which is called trans-signalling 38. Compared with IL-6R, sIL-6R lacks cytoplasmic and transmembrane regions, binds to IL-6 and activates downstream signalling via ubiquitously expressed gp130 on the cell surface 39, which may also contribute to the pathological process of NMOSD (Figure 1).

### Type I interferon (IFN-I)

Type I interferons (IFN-Is), which are group of polypeptides associated with intracellular antimicrobial programmes, are crucial in mediating innate and adaptive immune responses 40. It has been determined that the chronic production of IFN-Is in the CNS is the causal factor driving the development of NMOSD (Figure 1). The IFN-I family consists of many subtypes, the human IFN-I family includes IFN-α, IFN- β, IFN- ε, IFN-κ, and IFN-ω, and the mouse origin includes IFN-α, IFN-β, IFN-ε, IFN-κ, and IFN-ζ 25, 41. The serum level of IFN-α in patients with NMOSD is increased, which is positively correlated with disease severity 42. IFN-β is widely used for treating MS, but it has no treatment effect in NMOSD, and even aggravates the severity of the disease 43.

Almost every CNS-resident cell can express and secrete IFN-I, including microglia, neurons, astrocytes and endothelial cells. IFNAR1 and IFNAR2, the IFN-I receptor genes, are also widely expressed in the CNS 25. Therefore, IFN-I binds to IFNAR and activates downstream signalling which may regulate 2'5'-OAS, CD86, MHC-Ⅰ and MHC-Ⅱ, and this effect may be widespread in different cell types of the CNS 25 (Figure 1). Importantly, the response of microglia to IFN-I is more pronounced than that of astrocytes or neurons 44, indicating that microglia may account for the major cell population that responds to IFN-I in NMOSD. A recent study determined that IFN-β treatment exacerbates the severity of NMOSD and induces microglial activation, as indicated by the expansion of a CD11c^+^ subset of microglia, suggesting that IFNI-activated microglia play a pathologic role in NMOSD 43.

## Factors secreted by activated microglia are involved in the pathogenesis of NMOSD

### Complement

AQP4 is mainly distributed on astrocyte foot processes located in the blood-brain barrier. AQP4-specific antibody (NMO-IgG) supports the cooperation between the cellular and humoral arms of adaptive immunity in NMOSD pathogenesis 45 which is capable of activating complement. Complement proteins are involved in astrocyte destruction and secondary demyelination during the pathological process of NMOSD 27. Therefore, NMOSD is also considered a complement-mediated astrocytopathy.

**Figure 2:**
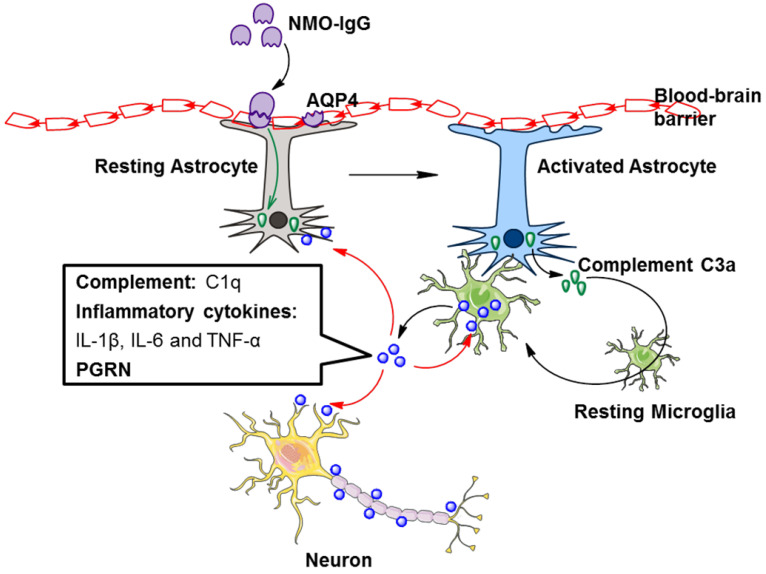
** Factors secreted by activated microglia are involved in the pathogenesis of NMOSD.** NMO-IgG binds to AQP4 on astrocytes, resulting in astrocyte activation. Activated astrocytes secrete complement C3a, which binds with C3aR on resting microglia, leading to microglial activation. The secretion of complement C1q, inflammatory cytokines and PGRN by activated microglia is increased. These secretions further promote microglial activation in an autocrine manner and facilitate localized injury to neurons, astrocytes and oligodendrocytes in a paracrine manner. AQP4, aquaporin 4; NMO-IgG, neuromyelitis optica immunoglobulin; PGRN, progranulin.

It is accepted that NMO-IgG can induce massive production of C3 by astrocytes 46, and C3a receptor (C3aR) is mostly expressed on microglia 47. Whether in healthy or pathological conditions, astrocytes and microglia coordinate their functions 48. We may speculate that astrocytes could promote microglial activation through the C3 cleavage product C3a. A recent study showed that NMO-IgG binds to AQP4 on astrocyte endfoot processes, resulting in AQP4 internalization, decreased cell surface AQP4 expression, and astrocyte activation 30. Elevated C3 produced by activated astrocytes is cleaved to form C3a and C3b. Secreted C3a binds with C3aR on resting microglia, which promotes microglial activation, the production of C1q, and the convergence towards astrocytes 30. C1q derived from activated microglia facilitates localized injury to neurons and oligodendrocytes 30 (Figure 2). In addition, C1q may activate the classic complement cascade, leading to sustained CNS tissue damage in NMOSD 26.

In addition to complement, TGF-β derived from astrocytes induces neuronal release of complement, which ultimately acts upon microglia 48. Astrocyte-derived IL-1 could change the permeability of the blood-brain barrier, accordingly allowing microglial activation 48. Astrocytes and microglia directly affect each other via molecules such as IL-1a, TNF, and IL-33 48. Hence, astrocyte-microglial communication may be a target of NMOSD therapy (Figure 2).

### Inflammatory cytokines

Microglial activation is a two-edged sword in NMOSD. Microglial activation may lead to demyelination 49, while it has also been reported to contribute to remyelination 50. Microglia is capable to secrete both pro- and anti-inflammatory cytokines in response to external stimuli 51. Correspondingly, activated microglia can be mainly polarized into two functional phenotypes: M1 and M2 phenotype 52-55.

In the initial stage of CNS diseases, activated microglia are dominated by the M2 phenotype, which is related to the release of several anti-inflammatory cytokines, including IL-10, IL-4, IL-13 and TGF-β, and proinflammatory cytokine production is suppressed 56. Meanwhile M1 phenotype activation is connected with the release of proinflammatory cytokines, such as IL-1β, IL-6 and TNF-α, and an excess accumulation of these factors caused by chronic activation of microglia can result in damage to the surrounding neuronal cells 51. These proinflammatory cytokines are involved in the pathological process of NMOSD 57. For example, IL-1β induces neuromyelitis optica-like lesions in a *in vivo* study 58. Collectively, M1/M2 polarization might regulate microglial activation as a dual role in NMOSD. However, the regulatory role of inflammatory cytokines derived from microglial activation in NMOSD has not been deeply studied and might be a potential research field.

### Progranulin (PGRN)

Progranulin (PGRN), a secreted glycosylated protein, was originally considered to be a growth factor that regulates immune responses and cancer growth 59. PGRN has been reported to be highly enriched in microglia and neurons 60. Kimura et al. reported that CSF levels of PRRN in NMOSD patients are notably higher than those in MS patients and controls without inflammation 61. They also demonstrated that CSF levels of PGRN have a significant correlation with cell counts and protein levels in CSF. It also affects total spinal cord lesion length, which may be related to the severity of spinal cord inflammation in NMOSD 61. Furthermore, there is a positive correlation between IL-6 and PGRN levels in patients with NMOSD and MS 61. As IL-6 triggers PGRN expression in cancer studies 62, 63, IL-6 may serve as a crucial regulator of PGRN in response to microglial activation. It has also been indicated that PGRN enhances IL-6 expression in adipocytes 64. Therefore, CSF PGRN in NMOSD may be mainly derived from microglia and may have interactive regulation with IL-6.

A growing body of evidence suggests that PGRN functions as an autocrine neuroprotective factor by modulating neuroinflammation 59. PGRN-deficient mice are hypersusceptible to neuroinflammation and neuronal loss in response to the injury, and PGRN exhibits a neuroprotective effect by decreasing proinflammatory cytokines and increasing anti-inflammatory cytokines 60, 65-67. In view of this evidence and recent research advances on the role of PGRN in NMOSD, PGRN derived from activated microglia may provide negative feedback in NMOSD (Figure 2).

## Prospects and potential applications

Recently, microglia has been identified as potential modulators and targets for NMOSD diagnosis and therapy because of their important roles in neurological disorders.

### Regulation by noncoding RNAs

Although they are never translated into proteins, noncoding RNAs (ncRNAs) have been recognized as playing essential regulatory roles in the human CNS, as well as in neurological diseases 68. It has been indicated that microRNAs (miRNAs) are significantly altered in the whole blood of NMOSD patients when compared to MS patients^69^, ^70^, while long noncoding RNAs (lncRNAs), the another kind of ncRNA, are differentially expressed in peripheral blood mononuclear cells (PBMCs) of NMOSD patients compared to those of controls 71. Circulating miRNAs from whole blood, as well as serum exosomal miRNAs, might serve as potential biomarkers for the diagnosis and prognosis of NMOSD 72, 73, and several miRNAs are also capable of discriminating NMOSD from MS or neuropsychiatric systemic lupus erythematosus (NPSLE) 74, 75. It has been revealed that different kinds of ncRNAs (miRNAs 76, lncRNAs 77, circular RNAs (circRNAs) 78, etc.) are involved in the regulation of microglia in the pathogenesis of neurological diseases such as MS and epilepsy. The mechanisms include regulating gene expressions and microglia polarization, and acting as a molecular sponge. However, the relevance and mechanisms of microglia and ncRNAs in NMOSD remain unclear and deserve to be further investigated.

### Utilization of other novel technologies

Recently, many new technologies have been used to study NMOSD and other autoimmune neurological disorders. For example, single-cell sequencing technologies have been applied to investigate B cells and antibodies in the blood and CSF of NMOSD patients 79. Haematopoietic stem cell transplantation has been considered a possible option for treating patients with severe NMOSD 80. To reduce brain lesions and therapeutically treat patients with CSF1R-related leukoencephalopathy, haematopoietic stem cell transplantation could renew dysfunctional microglia 81. The effect and application of haematopoietic stem cell transplantation on microglia should be further investigated in patients with NMOSD. Although there are some limitations, such as immunogenic reactions and a lack of specificity of viral vectors, gene therapy has been applied with positive outcomes in neurological diseases, including MS 82. Insulin-like growth factor 1 (IGF1) gene therapy could target microglia, modify the inflammatory response and improve cognitive or motor deficits 83. Therefore, gene therapeutic approaches might be a promising choice to partially modulate the progression of NMOSD by targeting microglial activation. To identify new therapeutic and diagnostic approaches with safety and efficacy, these novel technologies should be utilized in future studies of microglial activation in NMOSD.

## Conclusion

Although the functions and mechanisms of microglia remain under investigation, the activation of these immunomodulatory cells attracts the attention of researchers. Cytokines such as IL-6 and the IFN-I family, the complement system and PGRN are involved in the pathogenesis of NMOSD. M1 or M2 polarization might regulate microglial activation in NMOSD. Combined studies with ncRNAs, as well as novel technologies such as single-cell sequencing, stem cell therapy and gene therapy, are promising options for investigating the microglial activation in NMOSD.
